# Personality Factors Crucial in Internalized Stigma Understanding in Psychiatry

**DOI:** 10.3390/healthcare9040456

**Published:** 2021-04-13

**Authors:** Dorota Szcześniak, Agnieszka Kobyłko, Marta Lenart, Maciej Karczewski, Agnieszka Cyran, Piotr Musiał, Joanna Rymaszewska

**Affiliations:** 1 Department of Psychiatry, Wroclaw Medical University, 50-367 Wroclaw, Poland; dorota.szczesniak@umed.wroc.pl (D.S.); marta.lenart@umed.wroc.pl (M.L.); agnieszka.cyran@umed.wroc.pl (A.C.); piotr.musial@umed.wroc.pl (P.M.); joanna.rymaszewska@umed.wroc.pl (J.R.); 2 Department of Applied Mathematics, Wroclaw University of Environmental and Life Sciences, 50-357 Wroclaw, Poland; maciej.karczewski@manteion.pl

**Keywords:** internalized stigma, personality traits, intrapersonal factors, severe mental illness, ISMI scale

## Abstract

*Objective*: The main purpose of this research was to establish the relationship between personality traits and internalized stigma in individuals living with severe mental illness. Additionally, the study aimed to identify individual differences that could be used to develop the theoretical socio-cognitive-behavioral equation model of internalized stigma. *Methods*: A total of 114 patients with diagnosis of nonorganic psychotic disorder or uni- or bipolar affective disorder took part in this study. The Internalized Stigma of Mental Illness (ISMI) scale, Eysenck Personality Questionnaire Revised (EPQ-R) and NEO Five-Factor Inventory (NEO-FFI) were administrated among all participants. *Results*: Patients presenting higher levels of neuroticism scored higher on the ISMI scale. Otherwise, those with higher levels of extraversion, openness to experience and conscientiousness had lower ISMI scores. With the use of multivariate linear regression, neuroticism, openness to experience and conscientiousness showed the strongest associations with internalized stigma. *Conclusions*: Intrapersonal factors such as personality traits might explain individual differences in responses to the stigmatization process. Moreover, sociodemographic conditions such as the place of residence and level of education can play a mediating role in reducing the level of internalized stigma. Adequate psychosocial interventions should consider demographics and personality traits when engaging patients with mental illnesses in activities aimed at understanding and accepting the disorders.

## 1. Introduction

Stigma has been researched in the context of many attributes, health conditions and social groups [1,2,3,4,5,6,7]. Regardless of culture or country, people living with mental disorders are more prone to stigma in society compared to other patient populations. According to Świtaj, these people are perceived as different, worse or even “not fully human”, which results in a multistage process of exclusion [8]. While this problem is not a new phenomenon, community psychiatry has only recently taken steps to emphasize the reintegration of this group into society.

It is assumed that stigma has three dimensions: social, structural and internalized [9]. A recent study in Poland showed that people living with mental illness experience a high sense of discrimination and social isolation. Moreover, the exclusion was noticeable in various spheres of life, and symptoms of discrimination were even displayed by health professionals [10]. The diagnosis of a mental illness often causes a specific social reaction towards the patient, e.g., isolation, devaluation or even excessive, unnecessary care. Such negative responses may, unfortunately, lead to the internalization of stigma, manifested by reduced self-acceptance, a sense of mismatch with social norms and identity change, as well as behavior consistent with stereotypes [11]. Our previous study found that severe mental illnesses such as psychosis and affective disorders are associated with mild levels of internalized stigma, as confirmed by studies from other countries. Duration of the disease and type of diagnosis turned out to be related to the level of internalized stigma among mental health patients [12]. However, these factors are insufficient for a comprehensive analysis of the phenomenon of stigma. To this end, the importance of personality traits in mediating between social stigma and its internalization began to be explored [13]. Personality differences are defined by “habitual patterns of thought, emotion and behavior, which are relatively stable over time and differ across individuals” [14]. These individual differences may explain why some people with mental illness are more prone to stigma, while others are able to develop adaptive coping strategies.

When looking for an answer to the question about the factors determining the feeling of the stigma of mental illness, one should undoubtedly consider the social environment, along with the level of awareness and knowledge about the problem, as well as personal life experience. On the other hand, the very personality of the person experiencing discrimination may play an important role.

The effectiveness of self-assessment methods in studying personality traits among patients with severe mental illnesses such as schizophrenia was suggested by Bell et al., while emphasizing the role of insight into the disease [15]. However, the relationship between personality factors and the degree of internalized stigma has not yet been studied. To fill this gap, this study aims to identify the association of the internalized stigma with personality traits, sociodemographic factors and health-related factors in severe mental illness. The results are intended to help understand the role of individual differences in the stigma process, with the aim of developing a “theoretical model of socio-cognitive-behavioral equations of internalized stigma [9]”.

## 2. Materials and Methods

### 2.1. Study Design

The present study is the second part of a cross-sectional study consisting of two phases. In the first phase, the relevance of the Internalized Stigmatization of Mental Illnesses (ISMI) scale in Polish society and the validity of the phenomenon of internalized stigmatization were assessed [12]. Phase II, described in this article, focused on the intrapersonal, sociodemographic and health-related factors associated with internalized stigma. The study protocol (No. 404/2014) was approved by the local Bioethical Committee.

### 2.2. Study Sample and Setting

The research was conducted from 2015 to 2017 in the three different psychiatric institutions in Poland. The study participants (*n* = 120) recruited were patients hospitalized for at least four weeks, and participants were recruited from both inpatient and day wards. The inclusion criteria for the study were as follows: (1) age range 18–70 years; (2) psychiatric diagnosis of psychotic disorder (PS, schizophrenia, schizoaffective disorder, persistent delusional disorder), depression (D, episode or unipolar affective disorder), or bipolar disorder (BD) according to the International Classification of Diseases (ICD-10) [16].

The exclusion criteria were as follows: (1) severe acute symptomatology of mental illness; (2) psychiatric diagnosis of comorbidities and substance abuse, organic brain disease, severe somatic disease or intellectual disability.

All participants received complete information about the study protocol, their anonymity and the possibility to resign at any time. All participants were volunteers, signed an informed consent form prior to the study enrollment and had the opportunity to ask questions or for support throughout the study. Finally, as a result of incomplete data, information on 114 patients was used in the statistical analysis.

### 2.3. Measuring Instruments

The following instruments were used:

- The sociodemographic questionnaire, which contains information about participants—sex, age, education, marital status and employment status.
- The original clinical questionnaire, which contains information about the type of diagnosis, type of hospitalization (inpatient or outpatient), duration of the disease, current pharmacotherapy, number of hospitalizations and occurrence and number of suicide attempts. The researcher completed the questionnaire after analyzing the patient’s medical records and after an interview with the patient.
- The Internalized Stigma of Mental Illness Scale (ISMI), which is a Polish version of the questionnaire commonly used to estimate the level of internalized stigmatization among people with mental illness, originally developed by Ritsher et al. [17]. The scale comprises 29 statements, divided into five domains. The scores range between 1 and 4. The method by Lysaker et al. [18] was used for interpretation: 1.00–2.00, minimal/no internalized stigma; 2.01–2.50, mild internalized stigma; 2.51–3.00, moderate internalized stigma; 3.01–4.00, severe internalized stigma. Reliability and validation of the ISMI have been established for various disorders and cultures [19]. Cronbach’s α coefficient of reliability for the original and Polish version was 0.85 [12] and for this sample was 0.80.
- NEO-Five Factor Inventory (NEO-FFI), which is a questionnaire used to measure personality traits included in the Big Five Model [20]: neuroticism, extraversion, openness to experience, agreeableness and conscientiousness. The respondent assesses the truthfulness of each of the 60 items on a five-point scale. The reliability of the Polish version of the questionnaire, measured by the Cronbach’s α, ranged from 0.68 to 0.82, depending on the domain of a given trait; for this sample, it was 0.66 to 0.78.
- Eysenck Personality Questionnaire-Revised (EPQ-R), which is a questionnaire used to measure types of personality according to Eysenck’s theory [21]. The tool consists of 106 items divided into six scales. Three of them—psychoticism, extraversion and neuroticism, relate to the basic dimensions of personality. The control lies scale allows assessment of the tendency towards better self-presentation (dissimulation). The reliability of the Polish version measured by Cronbach’s α was fully satisfactory for all scales, except for psychoticism. For this sample, Cronbach’s α was 0.68. The reliability of the questionnaire was 0.73 if the neuroticism scale was dropped, 0.81 if the extraversion scale was dropped, 0.80 if the psychoticism scale was dropped, 0.79 if the control lies scale was dropped, 0.66 if the additional addiction scale was dropped and 0.69 if the additional criminality scale was dropped. In this study, only the domain of psychoticism was used, which is not included in the NEO-FFI.

Based on the results of Bell et al. [15], the use of self-assessment questionnaire methods in the study of personality traits among patients diagnosed with severe mental disorders such as schizophrenia was considered justified.

### 2.4. Statistical Analysis

Descriptive statistics (mean and standard deviation) were used to calculate variables (demographic and clinical) and ISMI scores. The Mann–Whitney and Kruskal–Wallis pairwise tests and post hoc analysis were performed to analyze the relationship between internalized stigma scores and qualitative demographic or clinical variables. For quantitative variables, the Spearman correlation coefficient was calculated. To assess the independent effect of clinical and demographic variables on stigma, univariate linear regression and multivariate linear regression were performed. All analyses were performed in R for Windows, version 3.4.3 (R Foundation for Statistical Computing, Vienna, Austria) [22].

## 3. Results

### 3.1. Sample Description

Details were previously described by Szcześniak et al. [12]. Table 1 and Table 2 show the study sample characteristics (*n* = 114). The majority of participants were single females, with secondary education, receiving a pension. Forty-five percent of the research group were diagnosed with psychotic disorder, 39% were diagnosed with depressive disorder and 16% were diagnosed with bipolar affective disorder. The illness duration was on average 9.65 (SD = 8.69) years, and the mean number of hospitalizations was 5.95 (SD = 6.91). Fifty-eight percent of participants received outpatient treatment. Most of them were treated with antipsychotics (87%) and antidepressants (70%). Forty-six patients (40%) had a history of suicide attempts. According to psychiatric examination, 69% had insight into their illness. Moreover, based on the total ISMI scores, participants presented a mild level of internalized stigma, as well as presenting mild levels in the majority of ISMI subscales: stereotype endorsement, perceived discrimination, social withdrawal and stigma resistance. Only in the alienation subscale was the study sample characterized by a moderate level. There was no significant difference between males and females in ISMI total score and its subscales (Table 2).

### 3.2. Total Score of Internalized Stigma

The determinants and correlates of the ISMI score are presented in Table 3. According to the univariate linear regression analysis, patients with higher education had a significantly lower level of internalized stigma. Illness duration was the only significant health-related factor that correlated positively with the total ISMI score. Significant relationships were also found between various personality traits and the overall ISMI score. A lower level of internalized stigma was related to greater extraversion, openness to experience and conscientiousness. On the other hand, those with higher scores on the neuroticism scale had higher scores on the total ISMI. Due to the significant associations noted, a multivariate linear regression analysis was performed to discover the key components of internalized stigma (see variables in Table 4). According to the regression model, the strongest predictors of internalized stigma turned out to be neuroticism, openness to experience and conscientiousness. The place of residence was an almost statistically significant factor (*p* = 0.07). Participants living in small towns had higher ISMI scores. The model accounted for 45% of the variance of the internalized stigma.

### 3.3. Alienation

The level of alienation in the sample was not related to sociodemographic factors. However, health-related variables, such as illness duration and the diagnosis of depressive disorders, showed significant relationships with this subscale (Table 3). Participants with higher extraversion, openness to experience and conscientiousness presented lower levels of alienation. Moreover, higher scores on the neuroticism scale (inclination to feel negative emotions) and lower scores on the conscientiousness scale were found to be predictors of higher alienation, also confirmed by the multivariate linear regression analysis (Table 5). The model explained 36% of the variance of this subscale.

### 3.4. Stereotype Endorsement and Perceived Discrimination

The statistical analysis showed that receiving pension and illness duration were related to stereotype endorsement and perceived discrimination. On the other hand, higher education turned out to be a protective factor of these subscales. Among factors related to individual differences, those with higher scores on the neuroticism scale had higher scores in both domains. However, subjects who were more extroverted and conscientious were less likely to believe in stereotypes and perceive themselves as discriminated against. However, those who were more open to new experiences had a higher level on the social withdrawal subscale.

In the multivariate linear regression analysis, these stigma subscales differ from each other in terms of the possible predictors. Conscientiousness turned out to be the only predictor of stereotype endorsement (Table 5)—the more conscientious a person is, the less he accepts the stereotype. Age was an almost statistically significant factor (*p* = 0.0507). The older the age, the higher scores on the stereotype endorsement subscale. The model explained 27% of the variance of this subscale. Predictors of the perceived discrimination domain were related to both environmental (sociodemographic) and intrapersonal factors. Living in a small town determined higher scores in this domain, while higher education and conscientiousness determined lower scores. The model explained 26% of the variance of these two subscales.

### 3.5. Social Withdrawal

Based on the results of the statistical analysis, people with higher education and shorter illness duration were characterized by significantly lower social withdrawal. As in other domains, individual personality traits, such as neuroticism, extraversion and conscientiousness, were statistically significant factors. Interestingly, in the social withdrawal subscale, the level of openness to experience and the level of agreeableness do not play a significant role. On the other hand, the multivariate linear regression analysis showed that higher education, openness to experience and conscientiousness were significant protective factors of social withdrawal, while neuroticism was a positive predictor (the model explained 45% of the variance).

### 3.6. Stigma Resistance

When analyzing the results on this subscale, it is important to reverse the coding of each question—the higher the score, the lower the stigma resistance. Thus, univariate linear regression analysis and Spearman correlation (Table 3) showed that, as in other domains, stigma resistance was associated with higher education, extraversion and openness to experience. Neuroticism increased the risk of being less resistant to stigma. These results were confirmed in the multivariate linear regression model, in which the role of the intrapersonal traits such as neuroticism is evident. Openness to experience plays an almost significant role (*p* = 0.06) in stigma resistance. Furthermore, women had lower scores in the stigma resistance subscale. However, this model explained only 11% of the variance of this subscale.

## 4. Discussion

The aim of this study was to investigate the role of intrapersonal factors in the process of internalized stigma in severe mental disorders and, thereby, to gain knowledge that could be the basis for developing a holistic model of the phenomenon of internalized stigma in psychiatry.

The model proposed by Munoz et al., which combines social, cognitive and clinical factors, does not include the role of intrapersonal factors, as indicated by Margetic et al., stressing the importance of individual personality differences in mediating between social and internalized stigma [9,23]. Moreover, as described in Corrigan and Watson’s model, there are different ways of responding to stigma, largely due to situational factors [24]. The authors mention lowered self-esteem/self-efficacy, righteous anger and relative indifference. The dissimilarity of these reactions is undoubtedly related to not only the social circumstances or personal experiences of stigmatization but also the complex construct of the patient’s personality. Thus, in this study, three groups of factors were considered, namely sociodemographic, clinical and personality factors, in order to broaden the perspective and indicate the complexity of the phenomenon. Although previous studies showed the significance of these factors, they analyzed them separately [12,25,26]. Hence, the novelty of the current study was the combination of these factors in a single model to identify their interrelationships.

The obtained results illustrated the individual psychosocial profile of a person particularly at risk of internalized stigma. The most significant risk factors were prolonged duration of the disease and higher level of neuroticism, regardless of the psychiatric diagnosis. As it turned out, an important additional risk factor of perceived discrimination was living in a small town, which is probably related to a lower sense of privacy and limited openness to unknown situations. This relationship was noted not only in stigma associated with mental illness, but also in sexual minorities and other stigmatizing diseases such as HIV/AIDS [27,28].

The results showed that the strongest predictor of internalized stigmatization was neuroticism, commonly described with a tendency to experience negative emotions, i.e., anger, anxiety or sadness [29]. Long-term experience of negative emotions directly reduces the ability to think effectively, decide about oneself and deal with stressful situations. Therefore, it can be concluded that neuroticism is strongly related to the stressful assessment of stigma; as a result, neurotic people develop maladaptive coping strategies. Thus, the phenomenon of feedback becomes apparent when the internalization of the trauma results from the personality trait and, at the same time, deepens this trait through experiencing negative emotions. Neurotic people may be reluctant to seek help, and if they choose to ask for support, they may react with anxiety, hindering the recovery process. Hence, individual differences of patients should be taken into account when planning psychoeducational programs in mental health centers aimed at preventing stigmatization.

Important findings from this study include the evidence of potential protective factors in the process of internalized stigma. One of them is higher education, which is associated with significantly lower results in terms of the overall internalized stigma and all its subscales, which is consistent with the results of studies conducted in 13 European countries and in Africa [30,31]. However, in a study from the United States, such a compound was obtained only in the stereotype endorsement domain [32]. The simplest explanation is that higher education is often associated with greater awareness and knowledge about the disease, as well as a certain distance to the situation in which we find ourselves [12]. Hence, in order to compensate for these baseline differences in educational attainment, awareness-raising psychoeducation in the field of mental illness plays an important role at every stage of the disease. Moreover, gender has also turned out to be important in stigma resistance. According to the multivariate model, men scored higher on this subscale.

Among the personality factors, conscientiousness showed a potential protective role for overall ISMI score and its three subscales: stereotype endorsement, perceived discrimination and social withdrawal. The impulses that are an inseparable part of human life are controlled and regulated thanks to conscientiousness [33]. According to Steel et al., this trait is associated with better prosperity and quality of life; thus, highly conscientious people are happier and more satisfied with life [34]. It is concluded that these people develop positive strategies for adapting to the consequences of the disease. Such strategies are related to task-oriented thinking, organization and a disciplined attitude that can help them regulate their emotions and maintain a positive self-image.

Another important personality trait significantly related to ISMI total score, social withdrawal and level of resistance to stigma was openness to experience, suggesting that it may play a protective role. People who achieve high results in openness to experience are more creative, more curious about the world and not afraid of change [35]. Usually, such people are also more interested in gaining new knowledge [36], which is associated with greater awareness of their own situation and feelings, thus making it easier to accept the disease. Moreover, openness determines searching for knowledge on important topics (e.g., sources, symptoms, effects of the disease) and a greater tendency to look for facts, not relying on stereotypes. Perhaps this explains why high scores on openness to experience are associated with better resistance to stigma. People who do not believe in stereotypes and are aware of their condition are less likely to withdraw from society, which at the same time may explain the relationship with the lower level of social withdrawal in this group of patients. Moreover, open-minded people tend to be more tolerant and open to different lifestyles and cultures, and it may be easier for them to accept not only other people’s otherness but also, above all, their own [37]. Previous studies showed a negative relationship between openness to experience and stigma of severe mental illness [38]. If people with greater openness to experience are more willing to accept mental illness, and thus their disorder, they are most likely not as prone to internalizing the stigma.

## 5. Limitation of the Study

This study has several limitations. The relatively small sample size implies caution in generalizing the obtained conclusions. The results of a multivariate linear regression analysis on such a small group of patients may be inaccurate. Moreover, the associations were not adjusted for symptoms or disease severity. Hence, potential psychotic or depressive symptoms in patients may have influenced the results of internalized stigma. It is also worth emphasizing that self-assessment questionnaires require the patient’s readiness to share intimate problems and self-knowledge, which could also be a factor influencing the final results.

## 6. Conclusions

The aim of this study was to broaden the knowledge about internalized stigma and its relationship with individual differences in patients with mental disorders. The results highlighted the importance of personality traits, i.e., conscientiousness and openness to experience, as well as higher education in manifesting a lower level of internalized stigma. This sheds new light on the understanding of the complexity of stigma and its consequences, pointing to the interrelationships between personality, disease and the response to the stigmatization process. The knowledge about the influence of protective and intrapersonal factors on the perception of the disease and the internalization of stigma indicates a specific role of psychoeducation in both prevention and long-term treatment. Therefore, there is a need to assess the effectiveness of psychoeducational programs aimed at coping with emotions and their regulation in neurotic patients with mental illness, who are more prone to internalizing stigma. Moreover, considering individual personality traits and psychopathology and other potential factors in studying the response to stigmatization seems crucial to understanding the course and treatment of mental illness. Undoubtedly, strengthening the internal resources of patients based on their need to be open to new experiences, as well as increasing their awareness and knowledge about the disease, may contribute to shaping a sense of agency and acceptance of their own limitations, thus building their resistance to stigmatization.

## Figures and Tables

**Table 1:** Study sample characteristics (1).

Characteristics	Frequency	Percent
Gender		
Female	63	55
Male	51	45
Education		
Elementary	12	11
Vocational training	30	26
Secondary school	43	38
Higher education	22	19
Not completed	7	6
Employment status		
Unemployment	25	22
Retirement	11	10
Pension	47	41
Hired	24	21
Not completed	7	6
Marital status		
Single	53	46
Married	33	29
Divorced	16	14
Widowed	8	7
Not completed	4	4
Diagnosis		
Psychotic disorder	51	45
Depressive disorder	44	39
Bipolar affective disorder	19	16
Treatment		
Outpatient	67	58
Inpatient	44	38
Not completed	3	3
Pharmacological treatment		
Only antipsychotic drugs	21	23
Only antidepressant drugs	13	14
Only mood stabilizers	2	2
Only anxiolytic drugs	0	0
Antipsychotic drugs and antidepressant drugs	22	24
Antipsychotic drugs and mood stabilizers	16	17
Antipsychotic drugs and anxiolytic drugs	4	4
Antidepressant drugs and anxiolytic drugs	7	8
Antidepressant drugs and mood stabilizers	5	6
Anxiolytic drugs and mood stabilizers	1	1
More than two types of drugs	18	20
Suicide attempts		
Yes	46	40
No	64	56
Not completed	4	4
Insight		
Yes	79	69
No	23	20
No data	12	11

**Table 2:** Study sample characteristics (2).

Gender	Statistical Measure	Age	ISMI Total	Alienation	Stereotype Endorsement	Perceived Discrimination	Social Withdrawal	Stigma Resistance
	Mean	42.46	2.32	2.62	2.08	2.30	2.33	2.37
	(SD)	(14.09)	(0.52)	(0.78)	(0.59)	(0.68)	(0.75)	(0.57)
Female	Mean		2.31	2.69	2.04	2.23	2.35	2.31
	(SD)		(0.51)	(0.79)	(0.58)	(0.68)	(0.76)	(0.63)
Male	Mean		2.34	2.54	2.12	2.39	2.31	2.45
	(SD)		(0.53)	(0.77)	(0.62)	(0.67)	(0.75)	(0.49)

Notes: SD—standard deviation; ISMI—The Internalized Stigma of Mental Illness Scale.

**Table 3:** Determinants of internalized stigma and its subscales among patients with severe mental illness using Spearman correlation for quantitative variables and univariate linear regression analysis for qualitative variables.

Factors	Determinants	ISMI Total	Alienation	Stereotype Endorsement	Perceived Discrimination	Social Withdrawal	Stigma Resistance
Sociodemographic factors	Age	NS	NS	NS	NS	NS	NS
	Higher education	b = −0.52 SE = 0.18 *p* < 0.005	NS	b = −0.42 SE = 0.18 *p* < 0.05	b = −0.42 SE = 0.18 *p* < 0.05	b = −0.59 SE = 0.18 *p* < 0.005	b = −0.59 SE = 0.18 *p* < 0.005
	Annuity	NS	NS	b = 0.33 SE = 0.15 *p* < 0.05	b = 0.33 SE = 0.15 *p* < 0.05	NS	NS
	Gender	NS	NS	NS	NS	NS	NS
Health-related factors	Illness duration	r = 0.3 *p* = 0.00483	r = 0.27 *p* = 0.00802	r = 0.3 *p* = 0.00483	r = 0.25 *p* = 0.01094	r = 0.27 *p* = 0.00802	NS
	Depressive disorders	NS	b = 0.26 SE = 0.13 *p* < 0.05	NS	NS	NS	NS
Intrapersonal factors	Psychoticism (EPQ-R)	NS	NS	NS	NS	NS	NS
	Neuroticism (NEO-FFI)	r = 0.55 *p* < 0.0001	r = 0.49 *p* < 0.0001	r = 0.36 *p* = 0.00024	r = 0.42 *p* < 0.0001	r = 0.54 *p* < 0.0001	r = 0.28 *p* = 0.00367
	Extraversion (NEO-FFI)	r = −0.54 *p* < 0.0001	r = −0.48 *p* < 0.0001	r = −0.42 *p* < 0.0001	r = −0.28 *p* = 0.00421	r = −0.48 *p* < 0.0001	r = −0.27 *p* = 0.00603
	Openness toexperience (NEO-FFI)	r = −0.41 *p* < 0.0001	r = −0.33 *p* = 0.00126	r = −0.32 *p* = 0.00143	NS	r = −0.42 *p* < 0.0001	r = −0.23 *p* = 0.02436
	Agreeableness (NEO-FFI)	NS	NS	NS	NS	NS	NS
	Conscientiousness (NEO-FFI)	r = −0.51 *p* < 0.0001	r = −0.51 *p* < 0.0001	r = −0.43 *p* < 0.0001	r = −0.36 *p* = 0.00021	r = −0.53 *p* < 0.0001	NS

Notes: *p*-value adjusted for multiple testing using Benjamini–Hochberg correction for Spearman correlation; NS, not significant; r—Spearman correlation; b—unstandardized beta in linear regression; SE—standard error; ISMI—The Internalized Stigma of Mental Illness Scale; EPQ-R—Eysenck Personality Questionnaire-Revised; NEO-FFI—NEO-Five Factor Inventory.

**Table 4:** Multivariate linear regression analysis with internalized stigma total score as an independent variable.

Regressor	b	SD	*t*	*p*-Value	Measures
Intercept	−0.131	0.095	−1.379	NS	*F-statistic* 7.807 *Multiple-R* 0.53 *Adjusted R-squared* 0.45
Gender	−0.074	0.081	−0.904	NS	
Age	0.148	0.086	1.731	NS	
Primary education	−0.055	0.189	0.292	NS	
Secondary education	−0.029	0.128	−0.223	NS	
Higher education	−0.274	0.163	−1.680	NS	
Large city	−0.141	0.175	−0.808	NS	
Small city	0.225	0.122	1.842	0.07	
Medium city	−0.280	0.177	−1.583	NS	
Psychoticism (EPQ-R)	−0.093	0.085	−1.096	NS	
Neuroticism (NEO-FFI)	0.331	0.102	3.229	<0.005	
Openness to experience (NEO-FFI)	−0.254	0.086	−2.936	<0.005	
Conscientiousness (NEO-FFI)	−0.282	0.105	−2.674	<0.05	

Notes: NS, not significant; SD—standard deviation; b—unstandardized beta in linear regression; *t*—t-statistic; EPQ-R—Eysenck Personality Questionnaire-Revised; NEO-FFI—NEO-Five Factor Inventory.

**Table 5:** Multivariate linear regression analysis with internalized stigma scores on all subscales as independent variables.

ISMI Subscales	Regressor	b	SD	*t*	*p*-Value	Measures
Alienation	Intercept	−0.126	0.102	−1.237	0.2194	*F-statistic* 5.697 *Multiple-R* 0.45 *Adjusted R-squared* 0.36
	Gender	0.06557	0.08719	0.752	0.45418	
	Age	0.15611	0.09155	1.705	0.09186	
	Primary education	0.05549	0.20212	0.275	0.78433	
	Secondary education	0.07005	0.13671	0.512	0.60972	
	Higher education	−0.21662	0.17460	−1.241	0.21819	
	City of >250,000 inhabitants	−0.18550	0.18718	−0.991	0.32451	
	City of ≤50,000 inhabitants	0.07894	0.13061	0.604	0.54722	
	City of 50,000–250,000 inhabitants	−0.18773	0.18950	−0.991	0.32470	
	Psychoticism (EPQ-R)	−0.12287	0.09124	−1.347	0.18171	
	Neuroticism (NEO-FFI)	0.33172	0.10962	3.026	0.00329 *	
	Openness to experience (NEO-FFI)	−0.14283	0.09250	−1.544	0.12633	
	Conscientiousness (NEO-FFI)	−0.30093	0.11289	−2.666	0.00921 *	
Stereotype endorsement	Intercept	−0.116	0.110	−1.051	0.2965	*F-statistic* 4.06 *Multiple-R* 0.37 *Adjusted R-squared* 0.27
	Gender	−0.14973	0.09424	−1.589	0.11586	
	Age	0.19618	0.09895	1.983	0.05068 *	
	Primary education	−0.07509	0.21845	−0.344	0.7319	
	Secondary education	−0.00727	0.14776	−0.049	0.96088	
	Higher education	−0.18988	0.18871	−1.006	0.3172	
	City of >250,000 inhabitants	−0.152063	0.2023	−0.752	0.45435	
	City of ≤50,000 inhabitants	0.164698	0.141165	1.167	0.24663	
	City of 50,000–250,000 inhabitants	−0.171082	0.20481	−0.835	0.40591	
	Psychoticism (EPQ-R)	−0.005344	0.09861	−0.054	0.95691	
	Neuroticism (NEO-FFI)	0.093117	0.118473	0.786	0.43409	
	Openness to experience (NEO-FFI)	−0.173824	0.099974	−1.739	0.08575	
	Conscientiousness (NEO-FFI)	−0.365357	0.122016	−2.994	0.00361 *	
Perceived discrimination	Intercept	−0.15081	0.11205	−1.346	0.182	*F-statistic* 3.925 *Multiple-R* 0.36 *Adjusted R-squared* 0.26
	Gender	−0.005621	0.09598	−0.586	0.5597	
	Age	−0.05076	0.10078	−0.1008	0.6158	
	Primary education	0.1841	0.22249	0.827	0.4103	
	Secondary education	−0.11616	0.15049	−0.772	0.4423	
	Higher education	−0.46103	0.19220	−2.399	0.0187 *	
	City of >250,000 inhabitants	−0.22857	0.20605	−1.109	0.2705	
	City of ≤50,000 inhabitants	0.31859	0.14378	2.216	0.0294 *	
	City of 50,000–250,000 inhabitants	−0.208	0.2086	−0.997	0.3216	
	Neuroticism (NEO-FFI)	0.22738	0.12067	1.884	0.0630	
	Conscientiousness (NEO-FFI)	−0.28618	0.12427	−2.303	0.0238 *	
Social withdrawal	Intercept	−0.053	0.096	−0.555	0.5803	*F-statistic* 7.69 *Multiple-R* 0.52 *Adjusted R-squared* 0.45
	Gender	0.01664	0.08217	0.203	0.84001	
	Age	0.10561	0.08628	1.224	0.22435	
	Primary education	0.34706	0.19047	1.822	0.072	
	Secondary education	−0.04548	0.12883	−0.353	0.72497	
	Higher education	−0.41769	0.16454	−2.539	0.01298 *	
	City of >250,000 inhabitants	0.09458	0.17639	0.536	0.59324	
	City of ≤50,000 inhabitants	0.14172	0.12308	1.151	0.25284	
	City of 50,000–250,000 inhabitants	−0.29114	0.17858	−1.630	0.10678	
	Psychoticism (EPQ-R)	−0.08194	0.08598	−0.953	0.34333	
	Neuroticism (NEO-FFI)	0.31140	0.10330	3.015	0.00340 *	
	Openness to experience (NEO-FFI)	−0.24876	0.08717	−2.854	0.00544 *	
	Conscientiousness (NEO-FFI)	−0.30076	0.10639	−2.827	0.00587 *	
Stigma resistance	Intercept	0.018	0.117	0.158	0.8751	*F-statistic* 2.056 *Multiple-R* 0.227 *Adjusted R-squared* 0.11
	Female	−0.21093	0.10016	−2.106	0.0382 *	
	Age	0.11194	0.10517	1.064	0.2902	
	Primary education	−0.40950	0.23217	−1.764	0.0814	
	Secondary education	−0.00320	0.15704	−0.020	0.9838	
	Higher education	0.52169	0.20056	2.601	0.0110	
	Psychoticism (EPQ-R)	0.13800	0.10480	1.317	0.1915	
	Neuroticism (NEO-FFI)	0.29603	0.12591	2.351	0.0211 *	
	Openness to experience (NEO-FFI)	−0.20072	0.10625	−1.889	0.0623 *	
	Conscientiousness (NEO-FFI)	0.17936	0.12968	1.381	0.1703	

Notes: * Statistically significant *p*-value; SD—standard deviation; b—unstandardized beta in linear regression; *t*—t-statistic; ISMI—The Internalized Stigma of Mental Illness Scale; EPQ-R—Eysenck Personality Questionnaire-Revised; NEO-FFI—NEO-Five Factor Inventory.

## Data Availability

The data is presented within the article.
